# Anti-leishmanial activity of *Eleutherine plicata* Herb. and predictions of isoeleutherin and its analogues

**DOI:** 10.3389/fchem.2024.1341172

**Published:** 2024-03-06

**Authors:** Kelly Cristina Oliveira de Albuquerque, Andreza do Socorro Silva da Veiga, Fernando Tobias Silveira, Marliane Batista Campos, Ana Paula Lima da Costa, Ananda Karolyne Martins Brito, Paulo Ricardo de Souza Melo, Sandro Percario, Fábio Alberto de Molfetta, Maria Fâni Dolabela

**Affiliations:** ^1^ Biotechnology and Biodiversity Postgraduate Program (BIONORTE), Federal University of Pará, Belém, PA, Brazil; ^2^ Pharmaceutical Innovation Postgraduate Program, Federal University of Pará, Belém, PA, Brazil; ^3^ Leishmaniasis Laboratory, Evandro Chagas Institute, Ananindeua, PA, Brazil; ^4^ Laboratory of Molecular Modeling, Institute of Exact and Natural Sciences, Federal University of Pará, Belém, PA, Brazil; ^5^ Faculty of Pharmacy, Federal University of Pará, Belém, PA, Brazil; ^6^ Pharmaceutical Sciences Postgraduate Program, Federal University of Pará, Belém, PA, Brazil

**Keywords:** isoeleutherin, trypanothione reductase, antiamastigote activity, naftoquinones, medical plant

## Abstract

**Introduction:** Leishmaniasis is caused by protozoa of the genus *Leishmania*, classified as tegumentary and visceral. The disease treatment is still a serious problem, due to the toxic effects of available drugs, the costly treatment and reports of parasitic resistance, making the search for therapeutic alternatives urgent. This study assessed the *in vitro* anti-leishmanial potential of the extract, fractions, and isoeleutherin from *Eleutherine plicata*, as well as the *in silico* interactions of isoeleutherin and its analogs with Trypanothione Reductase (TR), in addition to predicting pharmacokinetic parameters.

**Methods:** From the ethanolic extract of *E. plicata* (EEEp) the dichloromethane fraction (FDEp) was obtained, and isoeleutherin isolated. All samples were tested against promastigotes, and parasite viability was evaluated. Isoeleutherin analogues were selected based on similarity in databases (ZINK and eMolecules) to verify the impact on structural change.

**Results and Discussion:** The extract and its fractions were not active against the promastigote form (IC_50_ > 200 μg/mL), while isoeleutherin was active (IC_50_ = 25 μg/mL). All analogues have high intestinal absorption (HIA), cell permeability was moderate in Caco2 and low to moderate in MDCK. Structural changes interfered with plasma protein binding and blood-brain barrier permeability. Regarding metabolism, all molecules appear to be CYP3A4 metabolized and inhibited 2–3 CYPs. Molecular docking and molecular dynamics assessed the interactions between the most stable configurations of isoeleutherin, analogue compound 17, and quinacrine (control drug). Molecular dynamics simulations demonstrated stability and favorable interactions with TR. In summary, fractionation contributed to antileishmanial activity and isoleutherin seems to be promising. Structural alterations did not contribute to improve pharmacokinetic aspects and analogue 17 proved to be more promising than isoeleutherin, presenting better stabilization in TR.

## 1 Introduction

American tegumentary leishmaniasis (ATL), an infectious and non-contagious disease, is caused by protozoa of the genus *Leishmania*, with 7 species of parasites responsible for the disease in Brazil (WHO, 2020). Since 2015, a downward trend in ATL cases has been reported in 17 endemic countries in the Americas, and Brazil reported the highest number of cases registered in 2019 (OPAS, 2020).

For the treatment of ATL, pentavalent antimonials and amphotericin B are mainly used, drugs that have high toxicity, are parenterally administered, high-cost, and there are reports of lack of therapeutic response and parasite resistance to antimonials (Ponte-Sucre et al., 2017; Mann et al., 2021). Such resistance has been associated with an increase in the production levels of trypanothione proteins by the resistant parasites (Mukhopadhyay et al., 1996).

Trypanothione Reductase (TR) is an enzyme found in flagellated protozoa of the *Leishmania* genus and plays a crucial role in regulating the oxidative stress in these parasites. This NADPH-dependent flavoenzyme functions to control the concentration of reactive oxygen species and is, therefore, a potential target for research in the development of selective inhibitors (Mukherjee et al., 2020). Given the above, it is urgent to search for therapeutic alternatives that act on parasites resistant to antimonials, which can be administered orally and with less toxic potential.

**FIGURE 1:**
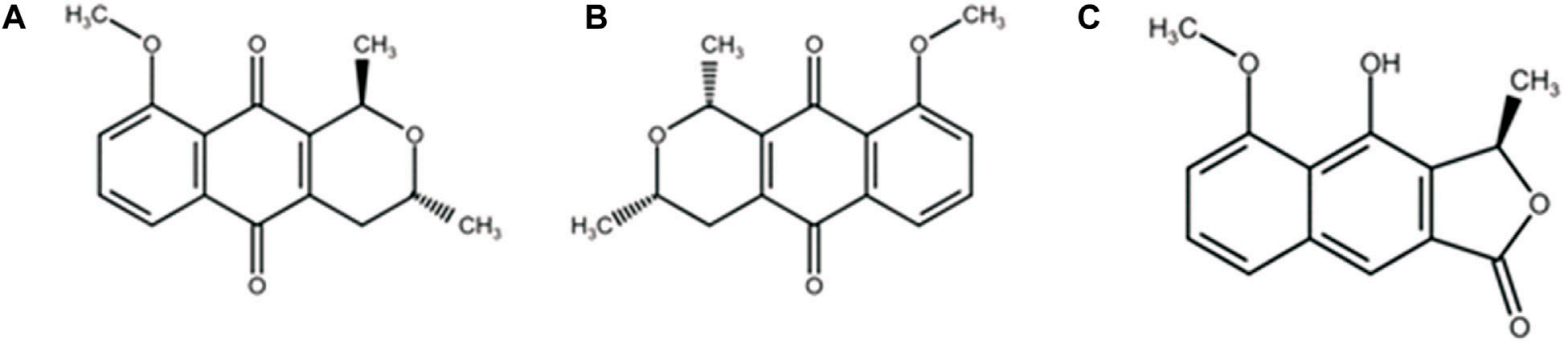
Compounds isolated from *Eleutherine plicata* Herb: **(A)** isoeleutherin; **(B)** eleutherin; **(C)** eleutherol.

Medicinal plants from the Amazon can be a promising source of leishmanicidal drugs, with some species being used to treat difficult-to-heal wounds (Silva et al., 2018). *Eleutherine plicata* Herb. is widely used in Amazonian folk medicine for the treatment of amoebiasis, liver diseases, parasitic infections, hemorrhages, anemia (Couto et al., 2016), as well as for the healing of superficial wounds and gastric ulcers (Villegas et al., 1997). Its main chemical constituents are naphthoquinones, including isoeleutherin (Figure 1A), eleutherin (Figure 1B), and eleutherol (Figure 1C), isolated from the bulb extract of this species (Figure 1) (Malheiros et al., 2015; Vale et al., 2020). Naphthoquinones eleutherin and isoeleutherin have been associated with the biological activities of the species (Paramapojn et al., 2008), and other studies have suggested their potential as potent trypanocidal and anticancer agents (Silva-Junior et al., 2019; Almeida et al., 2020; Castro et al., 2021b).

Toxicity assays in the *Allium cepa* model demonstrated eleutherin caused a higher percentage of chromosomal aberrations than isoeleutherin (12.5 μg/mL in 72 h). In the micronucleus assay, isoeleutherin showed low genotoxic potential, with a low frequency of micronuclei (Castro et al., 2021a).

Other study evaluated the genotoxicity of ethanol Extract (EEEp), Dichloromethane Fraction (FDCMEp) and isoeleutherin isolated from *Eleutherine plicata*, using the micronucleus test, is eleutherin was less genotoxic. Isoeleutherin and analogues were subjected to *in silico* toxicity prediction, and compounds free of toxicological risks (CP13, CP14, CP17, and isoeleutherin) were selected for molecular docking in Topoisomerase II. The structural changes suggest an increase in affinity with the TOPO II enzyme, observed in the increase in the amount of hydrogen bond interactions performed with amino acid residues of the active site (Albuquerque et al., 2023). The present study evaluated the leishmanicidal activity of isoeleutherin against the species of *Leishmania amazonensis*, associating *in silico* assays to search for new bioactive molecules.

## 2 Methodology

### 2.1 Plant material and isolation of isoeleutherin

Bulbs of *E. plicata* were collected in Tracuateua, Pará, Brazil (Lat. 1.1436°, Long. 46.9551°), a specimen was deposited at Museu Paraense Emílio Goeldi (MG 202631). The research project complies with national guidelines and international legislation, registered on the platform of the National System of Management and Genetic Heritage and Associated Traditional Knowledge (SISGEN), under registration number A49DEEE that grants license for collecting the species. The EEEp was obtained by macerating the dry powder of the bulbs (924 g) in ethanol (2 L for 7 days), subjected to fractionation in an open chromatographic column, using silica gel mesh (63–200 mm) as stationary phase and mobile solvents of increasing polarity (hexane, dichloromethane, ethyl acetate and methanol), obtaining the fractions: FHEp, FDEp, FAEEp, and FMEp, concentrated in a rotary evaporator. The FDEp was subjected to fractionation in thin layer chromatography on a preparative scale and showed four yellow spots with different retention factors, which were removed separately, being named Subfraction FA1, FA2, FA3, and FA4. From FA3, isoeleutherin was isolated, and identified by nuclear magnetic resonance (NMR) spectra, using a Bruker Advance DPX 400 MHz NMR spectrometer (Bruker Ascend).

### 2.2 Leishmanial activity against promastigotes and amastigotes of *L. amazonensis*

Inhibition of *Leishmania* growth was evaluated *in vitro* by cultivating promastigotes of *L. amazonensis* in stationary phase (5×10^6^ parasites) in the presence of the extract, fractions and isoeleutherin (200–3.125 μg/mL) in 96-well culture plates (Nunc, Nunclon^®^, Roskilde, Denmark), for 72 h at 26°C. Viability was assessed by measuring the cleavage of MTT [3-(4,5-dimethylthiazol-2-yl)-2,5-diphenyl tetrazolium bromide] (Sigma) (Ngure et al., 2009). Absorbances were measured using a multiwell scanning spectrophotometer (Molecular Devices, Spectra Max Plus, Canada) at 490 nm. Amphotericin B (AmpB) was used as a positive control (25–0.3906 μg/mL). The concentration of products required to inhibit 50% of the viability of *L. amazonensis* (IC_50_) was determined by applying a sigmoidal regression of the individual concentration response curves of the compounds. The data are representative of independent experiments, carried out in triplicate, which presented similar results (Mota et al., 2011).

Murine macrophages (4 × 10^5^ cells) were seeded on round glass coverslips into 24-well culture plates (Nunc) in RPMI 1640 medium (Sigma and Aldrich), supplemented with 20% fetal bovine serum (FBS), 2 mM L-glutamine, penicillin 50 IU/mL, and streptomycin 50 μg/mL, pH 7.4. After 24 h of incubation at 35°C in 5% CO_2_, promastigotes of *L. amazonensis* in stationary phase were added to the wells (4 × 10^6^ parasites) to promote infection of macrophages, and the cultures were incubated for 4 h at 35°C in 5% CO_2_. Next, the free parasites were removed by extensive washing with RPMI 1640 medium, and the infected macrophages were quantified and treated with the extract, FA2, FA3, fraction FA4, and isoeleutherin (500–125 μg/mL, each) for 72 h at 35°C in 5% CO_2_. The negative control consisted of infected macrophages and culture medium. The positive control used AmpB (100–25 μg/mL). Then, coverslips were stained with Giemsa, and the percentage of inhibition of intramacrophage viability of *Leishmania* was determined by counting the number of amastigotes per 100 macrophages on each coverslip, under a light microscope (×100 magnification). The presented data were performed in triplicate, and the IC_50_ was determined using GraphPad Prism version 5.04 (Silva, 2005).

### 2.3 Prediction studies of physical-chemical and pharmacokinetic aspects

Free online platform PreADMET (https://preadmet.webservice.bmdrc.org/) was used to predict absorption, distribution, metabolism, and elimination properties. Molecular descriptors related to Lipinski’s rule of five (Lipinski et al., 2001) and Veber extensions (Veber et al., 2002), such as molar mass (MW), octanol/water partition coefficient (cLog P), number of hydrogen bond acceptor (HBA) and hydrogen bond donor (HBD), number of rotatable bonds (nRotb) and topological polar surface area (TPSA) were predicted in Molinspiration (https://www.molinspiration.com) (Molinspiration, 2023).

Bioavailability prediction considered Lipinski’s “Rule of Five”, where a good drug candidate will have molecular weight <500, partition coefficient (log P) < 5, no more than five hydrogen bond donors and ten acceptors of hydrogen bonding (Lipinski et al., 2001). In the pharmacokinetic studies, intestinal absorption (Human Intestinal Absorption = HIA) was evaluated, considering parameters in the range of 0%–20% (low absorption), 20%–70% (moderate absorption), >70% (high absorption; (Hou et al., 2007). Molecule permeability in Caco-2 and MDCK cells were considered high when presented values >70 nm/s, average of 4–70 nm/s and low <4 nm/s (Yee, 1997; Yazdanian et al., 1998; Balimane et al., 2000). For distribution analysis, values >90% indicate strong binding to albumin, while <90% binding will be moderate to weak (Sun et al., 2018). As for the ability to cross the blood-brain barrier, the following criteria were used: freely crosses BBB >2.0, moderately crosses BBB values between 2.0 and 0.1 and reduced crossing or not crosses <0.1 (Ajay and Murcko, 1999).

### 2.4 Molecular docking simulations

Molecular docking was used to explore the possible conformations of the ligand with the binding receptor, estimating the intensity of enzyme-ligand interaction (Meng et al., 2011). Isoeleutherin and the subset derived from isoeleutherin analogue compounds were prepared from SMILES files downloaded from the ZINC (Irwin et al., 2012) and eMolecules (www.emolecules.com) databases (eMolecules, 2023). The crystallographic structure of the TR enzyme was retrieved from the Protein Data Bank (PDB) under the code 2JK6 (Baiocco et al., 2009) with a resolution of 2.95Å, prepared using the Chimera program (Lang et al., 2009), removing water molecules, ligands and adding hydrogen atoms. Molecular docking simulations were performed using the GOLD 2020.1 program (Cambridge Crystallo-graphic Data Center—CCDC, Cambridge, United Kingdom), which uses a genetic algorithm to generate and select conformations of flexible compounds that bind to the receptor site of a protein (Jones et al., 1995).

Compounds were scored by applying the GoldScore scoring function with a 100% efficient search. The binding site was defined based on studies by Padey et al. (2016), at a 10Å sphere centered on the flavin-adenine dinucleotide ligand (FAD); (Pandey et al., 2017a; Pandey et al., 2017b). The methodology was validated through redocking, evaluated using the fconv 1.24 program (Neudert and Klebe, 2011). The procedure was carried out to evaluate the convergence of the results and to determine the smallest value of the root mean square deviation (RMSD), making it possible to select the best interactions of the crystallographic ligand in the complex formed. To analyze the hydrogen bonds and hydrophobic interactions between the selected ligands and the enzyme’s amino acids, the PoseView online server was used (Stierand et al., 2006), a tool that displays molecular complexes that incorporate a simple and easy-to-perceive arrangement of the interactions formed between ligands and amino acids (Stierand and Rarey, 2007). Quinacrine was used as a control drug, which was chosen because it is a small molecule like the ones studied and because it has activity on TR (Saravanamuthu et al., 2004).

### 2.5 Molecular dynamics simulations

Firstly, the calculation of electrostatic potential charge was performed for the structure obtained during docking using the Gaussian 03 program (Frisch et al., 2003), applying the Restricted Electrostatic Potential (RESP) (Bayly et al., 1993) associated with the Hartree-Fock methods (Fock, 1930) and HF/6-31G (d,p) base function (Hariharan and Pople, 1973). The protonation states of all amino acid residues present in the enzyme were determined at pH 7.0 using the Propka server (Olsson et al., 2011). Molecular dynamics simulations were conducted using the AMBER 18 program, implemented by the pmemd. CUDA module (Li et al., 2013). The general AMBER force field (GAFF) (Wang et al., 2004) was employed to describe ligands, and the MMFF99SB force field (Hornak et al., 2006) was used for enzyme amino acid residues. The enzyme-ligand complex was solvated with the TIP3P explicit solvent model in a cubic box with an edge length of 12 Å, with the inclusion of Clˉ counter-ions to achieve electrical neutrality in the system, using the tleap module included in the AMBER program (Jorgensen et al., 1983).

Minimizations and heating were carried out with a SANDER module (Case et al., 2005), the systems were fragmented into four stages of energy minimization. In the first phase, 25,000 minimization steps were performed, divided into 10,000 steps, performed with the steepest descent method and 15,000 with the conjugate gradient. The remaining three phases were performed in 10,000 minimization steps, using the same methodology for each step. Subsequently, the systems were gradually heated using the Langevin algorithm, with protein atoms subjected to a restriction constant of 25 kcal/mol. Å2, considering the NVT set from 0 K to 298 K (Loncharich et al., 1992).

Periodic boundary conditions were simulated using the Particle Mesh Ewald (PME) method, employed for long-range electrostatic interactions (Darden et al., 1993). Cutoff distances for the long-range and van der Waals interactions were set at 9 Å. After minimization and equilibrium of the system, during the period of 50 ns of DM simulation, an integration time of 2.0 fs was produced using the Verlet algorithm (Verlet, 1968), considering the NPT set adjusted to a temperature of 298 K and pressure of 1 atm for each enzymatic binding complexes, all bonds with hydrogen atoms were restricted using the SHAKE algorithm (Ryckaert et al., 1977). The CPPTRAJ module of AMBERTOOLS 18 (Roe and Cheatham, 2013) was used to carry out the structural analyzes of RMSD and B-factor. The RMSD calculation verified the stability of the systems in relation to the initial structure, and the application of the B-factor identified which amino acid residues were most flexible in the enzyme. The binding free energy calculation was performed in the last 10 ns, using the AMBERTOOLS 18 modules CPPTRAJ and MMPBSA. py (Miller et al., 2012). The single-path MM-PB(GB)/SA protocol considers identical conformation states of the protein-ligand complex, unbound protein and free ligand (Kollman et al., 2000; Massova and Kollman, 2000).

The binding free energy (ΔG_bind_) of the complexes was determined according to Eq. 1, where ΔH is the enthalpy term, TΔS represents the product between absolute temperature and entropy resulting from the conformations obtained in the DM simulation, ΔEMM consists of the energy obtained by molecular mechanics and ΔGbind, solv is the free energy of solvation. Entropy contributions to energy result from changes in translation, rotation, and vibration.

$${∆G}_{bind\;}=∆H-T∆S\approx {∆E}_{MM\;}+{∆G}_{bind,solv\;}-T∆S$$ (1)

The mechanical energies, represented by Eq. 2, are calculated involving the individual contributions of internal energy (ΔEint), electrostatic (ΔEele) and van der Waals (ΔEvdW).

$${∆E}_{MM\;}={∆E}_{\mathrm{int}\;}+{∆E}_{ele\;}+{∆E}_{vdw}$$ (2)

The sum to compose the internal energy, described in Eq. 3, contains the contributions of bond length (ΔEbond), bond angles (ΔEangle) and torsion angles (ΔEtorsion).

$${∆E}_{\mathrm{int}\;}={∆E}_{bond\;}+{∆E}_{angle\;}+{∆E}_{torsion}$$ (3)

Solvation free energy (ΔG_bonding, solv_) results from the sum of polar (ΔG_PB/GB_) and non-polar (ΔG_n-polar_) contributions. According to Eq. 4, the polar electrostatic contribution to the solvation free energy can be calculated by the Poisson-Boltzmann method (PB) or by the generalized methods of Born approximation (GB). ΔG_PB_ and ΔG_GB_ were calculated using the generalized Born model (igb = 2) (Onufriev et al., 2000) and in the MMPBSA method, considering the dielectric constants of solute (Ajay and Murcko, 1999) and solvent (Yazdanian et al., 1998; Sun et al., 2014).

$${∆G}_{bind,solv\;}={∆G}_{PB/GB\;}+{∆G}_{non-polar}$$ (4)

The nonpolar energy is estimated by the product of the surface tension γ with a value equal to 0.0072 kcal/mol Å^2^ and the surface accessible solvent area (SASA) according to Eq. 5.

$${∆G}_{n-polar}=\gamma ∙SASA$$ (5)

The interaction free energy that allows determining the individual contribution of all residues to the free energy of the complexes, obtained by the MMGBSA method, is described in Eq. 6, elucidating its importance in the active site of the enzyme (Alves et al., 2020).

$${∆G}_{ligand-residue}={∆G}_{vdW}+{∆G}_{ele}+{∆G}_{GB}+{∆G}_{SA}$$ (6)

The main residues that contribute to the total energy were visualized using the CHEWD plugin (Raza et al., 2019) of the Chimera program (Pettersen et al., 2004). The van der Waals (ΔG_vdW_) and electrostatic (ΔG_ele_) interactions between amino acid residues of the TR enzyme were determined by the SANDER module, implemented in the AMBER 18 program.

## 3 Results

### 3.1 Phytochemistry, leishmanicidal activity, and cytotoxicity

From the ethanolic extract (EEEp; yield 2.84%) the following fractions were obtained: hexane (FHEp; yield 3.1%), dichloromethane (FDEp; yield 19.6%), ethyl acetate (FAEEp; yield 10.2%) and methanol (FMEp; 61.9% yield). Isoeleutherin was isolated from FDEp and its identification is described by Borges et al. (2020).

*In vitro* anti-leishmanial assays were conducted to evaluate the effect of the samples (EEEp, FDEp, FAEEp, FMEp, and isoeleutherin) on promastigote and intracellular amastigote forms of *L*. *amazonensis*. The results indicated no activity for the extract and its fractions, with an inhibitory concentration of 50% (IC_50_) exceeding 200 μg/mL, while isoeleutherin demonstrated activity (IC_50_ = 25 μg/mL). Additionally, it is important to note that the concentrations of the samples used in this study were not cytotoxic to macrophages (CC_50_ > 500 μg/mL), similar to the control drug (amphotericin B; CC_50_ > 100 μg/mL). The EEEp, its fractions, and isoeleutherin showed CC_50_ greater than 500 μg/mL and IC_50_ greater than 200 μg/mL against *L. amazonensis*, which is why the selectivity index calculation was not possible.

**FIGURE 2:**
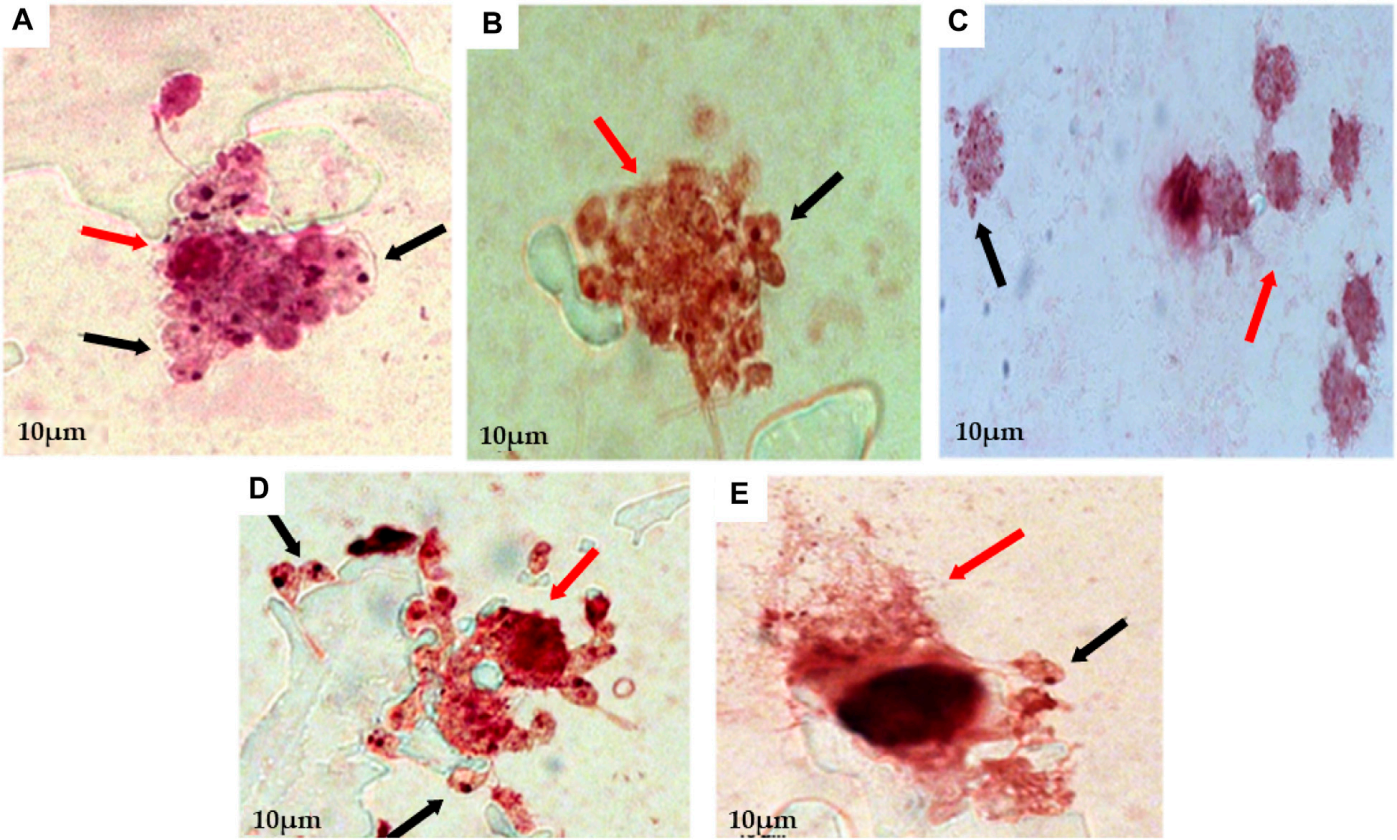
Assay for anti-amastigote activity, showing macrophages infected with amastigotes of *L. amazonensis* and then treated with 500 μg/mL of EEEp **(A)**, FDEp **(B)**, FAEp **(C)**, FMEp **(D)** and Isoeleutherin **(E)**. Red arrows indicate cell destruction (macrophages) and black arrows indicate the presence of amastigotes around the destroyed cell (×100 magnification).

**FIGURE 3:**
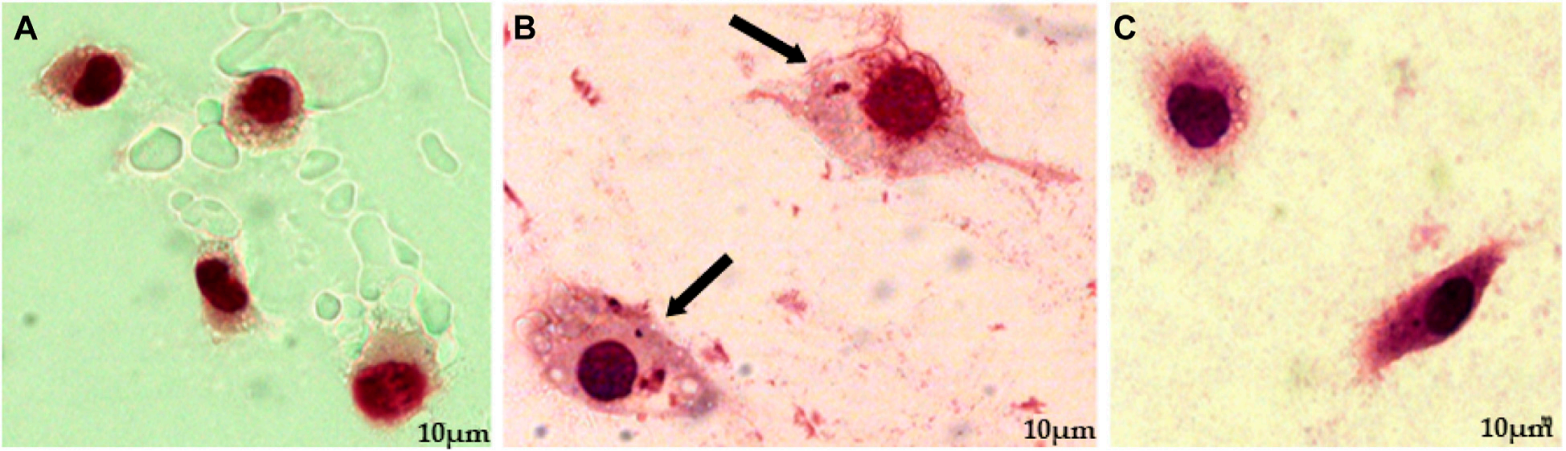
Anti-amastigote activity assay, showing uninfected macrophages, macrophages infected with *L. amazonensis* amastigotes, and subsequently treated with Amphotericin **(B)**. Caption: **(A)**. Uninfected macrophage control (×100 magnification); **(B)**. Macrophage control infected with *L. amazonensis* amastigotes (Negative control; ×100 magnification); **(C)**. Anti-amastigote action of Amphotericin B at a concentration of 100 μg/mL (Positive control; ×100 magnification). Arrows indicate the presence of amastigotes inside macrophages.

Then, macrophages infected with promastigote form were submitted to treatment with EEEp, FDEp, FAEEp, FMEp, and isoeleutherin (concentration of 500, 250 e 125 μg/mL). In Figure 2, amastigotes forms can be observed around the destroyed cells, exposed to EEEp (Figure 2A) and its fractions FDEp (Figure 2B), FAEEp (Figure 2C), FMEp (Figure 2D) and isoeleutherin (Figure 2E). The destruction of macrophages observed, likely caused by intracellular forms of *L. amazonensis*, since EEEp, its fractions, and isoeleutherin did not show cytotoxicity on macrophages. No reductions in the number of amastigotes were observed in infected cells when compared to the negative control (Figure 3).

Amastigote forms can be observed around the destroyed cells exposed to treatment with EEEp, FDEp, FAEp, FMEp, and isoeleutherin, highlighting that fractionation did not enhance the activity against amastigote forms of *L. amazonensis*, as the fractions and isoeleutherin remained inactive. The large number of promastigote forms around the destroyed macrophages indicates that they were unable to invade the cells, considering that these forms can survive in unfavorable conditions. Some of them assumed a more rounded shape, resembling amastigotes (Figure 2).

### 3.2 Prediction studies of pharmacokinetic and physicochemical aspects of isoeleutherin analogues

**FIGURE 4:**
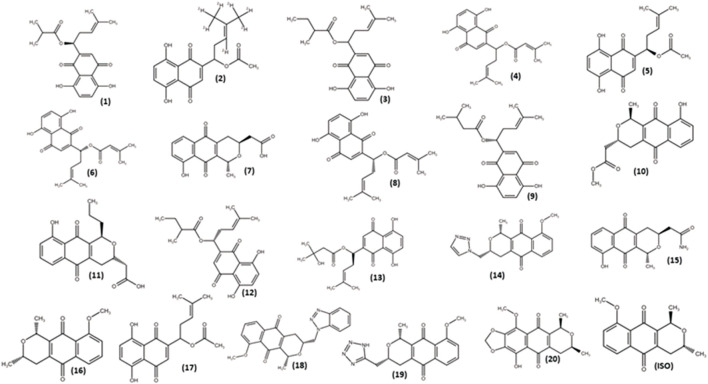
Isoeleutherin and its analogues.

Isoeleutherin (ISO; Figure 4) was employed as the starting molecule in the search for analogs through structural similarity in databases, with the aim of examining how structural modifications would impact physicochemical and pharmacokinetic properties. As a result of this process, 20 analogs (Figure 4) were identified and subsequently subjected to *in silico* prediction studies.

Isoeleutherin and its 20 analogues did not violate Lipinski’s rule, showing octanol-water partition coefficient (miLog *p* ≤ 5); Molecular Mass (MM) ≤ 500 g/mol, number of hydrogen bond acceptor groups (nHAG) ≤ 10 and number of hydrogen bond donor groups (nHDG ≤5) (Lipinski et al., 1997). The values obtained for the topological polar surface area (TPSA) parameters ≤140Å^2^; number of rotational bonds (Nrotb) ≤ 10 and molecular volume are within the limits established by Veber’s descriptors (Table 1) (Veber et al., 2002).

**TABLE 1:** Physicochemical aspects of isoeleutherin analogues.

Molecules	miLog P	MM	nHAG	nHDG	TPSA	Nrotb	Volume (cm^3^/mol)
Isoeleutherin	2.25	272.30	4	2	52.61	1	245.85
1	3.95	358.39	6	2	100.90	6	326.71
2	3.04	330.34	6	2	100.90	5	293.32
3	4.45	372.42	6	2	100.90	7	343.51
4	4.50	370.40	6	2	100.90	6	337.30
5	3.04	330.34	6	2	100.90	5	293.32
6	4.50	370.40	6	2	100.90	6	337.30
7	1.12	302.28	6	2	100.90	2	255.57
8	4.50	370.40	6	2	100.90	6	337.30
9	4.17	372.42	6	1	100.90	7	343.51
10	1.43	316.31	6	2	89.91	3	273.10
11	2.18	330.34	6	2	100.90	4	289.17
12	4.45	372.42	6	3	100.90	7	343.51
13	2.97	388.42	7	2	121.13	7	351.20
14	3.04	330.34	6	3	100.90	5	293.32
15	0.60	301.30	6	2	106.70	2	258.84
16	2.25	272.30	4	0	52.61	1	245.85
17	1.42	339.35	7	0	83.33	3	294.56
18	2.92	389.41	7	1	83.33	3	338.55
19	1.10	340.34	8	1	107.08	3	290.02
20	1.80	332.31	7	0	91.31	1	277.80

Octanol-water partition coefficient (miLog *p* ≤ 5); molecular mass (MM ≤ 500 g/mol), number of hydrogen bond acceptor groups (nHAG ≤10); number of hydrogen bond donor groups (nHDG ≤ 5); topological polar surface area (TPSA ≤ 140Å^2^); number of rotatable connections (Nrotb ≤ 10). Soucer: Lipinski et al., 1997; Veber et al., 2002.

The results suggest isoeleutherin and its analogues have moderate permeability in Caco2 and low to moderate in MDCK, whereas the permeability of isoeleutherin in MDCK is moderate. However, it is possible to observe that these compounds have high intestinal absorption (AIH). Isoeleutherine appears to bind to albumin moderately, with some structural alterations increasing the affinity for albumin. Only isoeleutherin and compound 18 freely crossed the BBB, structural alterations reduced the ability to cross the barrier. All compounds appear to be metabolized by CYP3A4, with variations in the magnitude of metabolism. Structural changes did not significantly impact the CYP inhibitory potential (Table 2).

**TABLE 2:** Pharmacokinetic aspects of isoeleutherin analogues.

Molecules	MDCK	Caco-2	HIA	PP	BBB	Metabolism CYP	Inhibition CYP (A)
Iso	Mod.	Mod.	High	Mod.	Free	3A4	2C19; 2C9; 34
1	Low	Mod.	High	High	Mod.	3A4	2C19; 2C9; 34
2	Mod.	Mod.	High	High	Red.	3A4*	2C19; 2C9; 34
3	Low	Mod.	High	High	Mod.	3A4	2C19; 2C9; 34
4	Low	Mod.	High	High	Mod.	3A4	2C19; 2C9; 34
5	Mod.	Mod.	High	High	Red.	3A4	2C19; 2C9; 34
6	Low	Mod.	High	High	Mod.	3A4	2C19; 2C9; 34
7	Mod.	Mod.	High	Mod.	Mod.	3A4*	2C19; 2C9; 34
8	Low	Mod.	High	High	Mod.	3A4	2C19; 2C9; 34
9	Low	Mod.	High	High	Mod.	3A4	2C19; 2C9; 34
10	Mod.	Mod.	High	Mod.	Mod.	3A4	2C19; 2C9; 34
11	Mod.	Mod.	High	High	Mod.	3A4*	2C19; 2C9; 34
12	Low	Mod.	High	High	Mod.	3A4	2C19; 2C9; 34
13	Low	Mod.	High	High	Mod.	3A4*	2C19; 2C9; 34
14	Low	Mod.	High	Mod.	Red.	3A4*	2C19; 2C9; 34
15	Mod.	Mod.	High	High	Red.	3A4*	2C19; 2C9; 34
16	Mod.	Mod.	High	Mod.	Free	3A4	2C19; 2C9; 34
17	Mod.	Mod.	High	Mod.	Mod.	3A4	2C19; 2C9; 34
18	Low	Mod.	High	High	Mod.	3A4	2C19; 2C9; 34
19	Mod.	Mod.	High	Mod.	Red.	3A4	2C9; 34
20	Mod.	Mod.	High	Mod.	Mod.	3A4	2C19; 2C9; 34

High Caco-2, and MDCK, permeability >70 nm/s, average of 4–70 nm/s and low <4 nm/s; Human Intestinal Absorption (HIA) low absorption 0%–20%, moderate 20%–70%, high >70%; Plasma protein binding (PP) strong >90%, moderate to weak <90%; blood-brain barrier (BBB) crosses freely >2.0, moderately 2.0–0.1, reduced or does not cross <0.1; Iso, isoeleutherin; Mod., moderate; Red., reduced; *weakly metabolized by the enzyme.

Soucer: Yee, 1997; Yazdanian et al., 1998; Balimane et al., 2000, Hou et al., 2007; Ajay and Murcko, 1999.

Therefore, it is important to evaluate the impact of structural alterations in different aspects, such as: physicochemical, pharmacokinetic and receptor binding. Among the isoeleutherin analogues, there were no significant alterations in physicochemical and pharmacokinetic aspects, except in distribution, where alterations in plasma protein binding and distribution to the CNS were observed.

### 3.4 Molecular docking and molecular dynamics

Molecular docking of the isoeleutherin and its 20 analogues were evaluated at the catalytic site of the TR enzyme (PDB 2JK6), performed at a distance of 10Å from the Flavin-Adenine Dinucleotide (FAD) cofactor, with RMSD values below 2Å, demonstrating that the redocking was successful according to literature data (Hevener et al., 2009). Connections that occurred in the active site of the enzyme were analyzed using the PoseView online server (Stierand et al., 2006), taking into account the interactions performed with residues Cys52, Cys57, His461’, and Glu466’, involved in the redox metabolism of Leishmania (Baiocco et al., 2009). The GoldScore scoring function was employed to predict the binding affinity of the most stable configuration, and the highest scores were considered to select the top 10 compounds with potential anti-leishmanial activity, as shown in Table 3.

**TABLE 3:** GoldScore values, hydrogen bonds, hydrophobic and π-π interactions of isoeleutherin and analogous obtained through Poseview.

Structure	GoldScore	Hydrogen bonds	Hydrophobic interactions	π-π interactions
Quinacrine	84.65	Thr335	Thr51, Gly56, Cys57	DT
Isoeleutherin	52.74	Ser14, Thr51, Ser162, Arg287	Gly161	DT
17	63.72	Tyr198, Cys57, Lys60	Thr51	DT
13	60.07	Lys60, Tyr198	Gly56, Ser178	DT
20	58.92	Ser14, Ser162	Cys57	DT
18	58.06	Lys60, Ser14	Ile199	DT
6	57.45	Lys60	Gly56	DT
1	57.10	Lys60	Gly56	DT
14	56.84	Lys60	Gly56, Ile199	DT
4	55.48	Thr51, Arg287, Cys57	Thr51	DT
15	54.93	Arg287, Ser162, Thr51	DT	DT
16	54.65	Lys60	Gly56	DT

DT- does not have.

**FIGURE 5:**
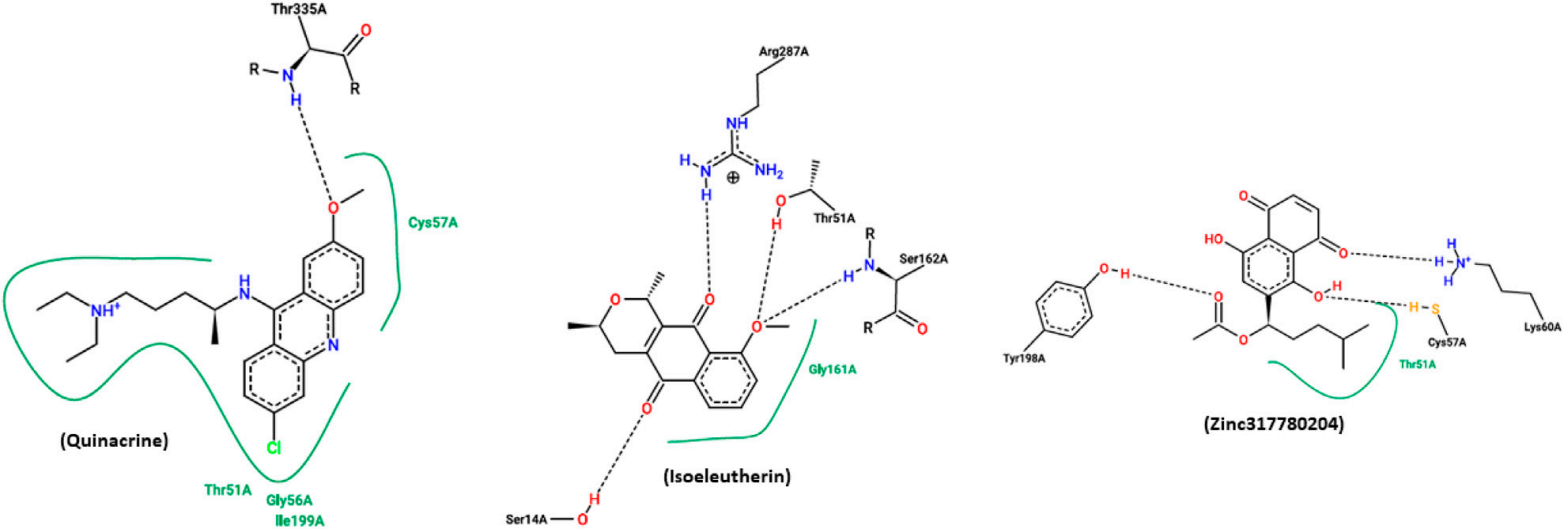
A figure generated by the online PoseView server, presenting the interactions resulting from docking simulations between the most stable configurations of quinacrine, isoeleutherin, and analogue compound 17 (Zinc317780204) with the enzyme TR. Hydrophobic interactions are indicated by continuous green lines, while hydrogen bonds are represented by dashed black lines.

Quinacrine is an effective and widely used antiparasitic drug with potential adverse effect. In this experiment, it was employed as the control drug. Compound 17 (Zinc317780204) was selected among the isoeleutherin analogues, based on the results found in molecular docking, showing that the molecule’s conformation interacts with the enzyme, through hydrophobic interactions with the amino acid residues Thr51 and hydrogen bonds with the Tyr198, Cys57, Lys60 residue. Isoeleuterin presented conformations with the enzyme by hydrophobic interactions with the residue Gly161 and hydrogen bonds with residues Ser14, Thr51, Ser162, and Arg287 (Figure 5).

**FIGURE 6:**
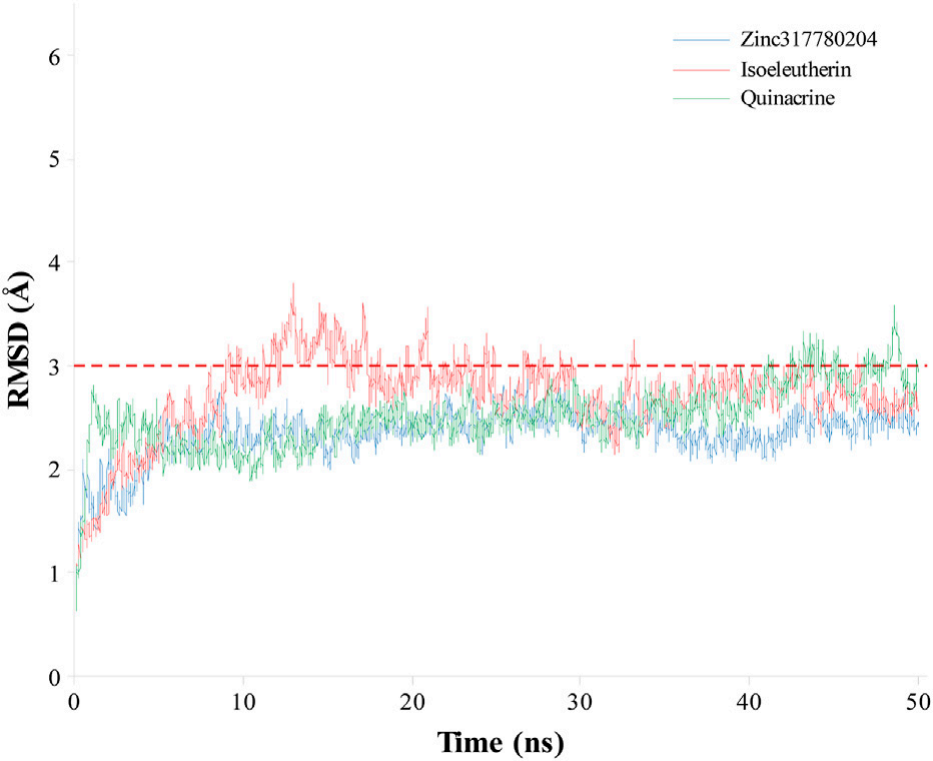
Graphical representation of RMSD (Å) values over time in the molecular dynamics simulation for isoeleutherin (red line), Zinc317780204 compound (blue line), and quinacrine (green line).

The coordinates obtained from the molecular docking simulation procedures, representing the most stable configurations of quinacrine, isoeleutherin, and the analogue compound (Zinc317780204), were subjected to 50 ns of Molecular Dynamics (MD) to assess the structural stability of the enzyme-ligand complexes through the analysis of RMSD values over time, as depicted in Figure 6. After the process was initiated, the RMSD values of the TR-ligand complexes fluctuated (1–4 Å) until reaching equilibrium (10 ns) and then remained stable throughout the simulation, with average values of 2.50 and a standard deviation of ±0.31 for quinacrine, 2.70 Å and a standard deviation of ±0.39 for isoeleutherin, and 2.33 Å and a standard deviation of ±0.23 for Zinc317780204. This indicates that the compounds remained within the enzyme cavity, showing minimal conformational changes in the complex structure.

**FIGURE 7:**
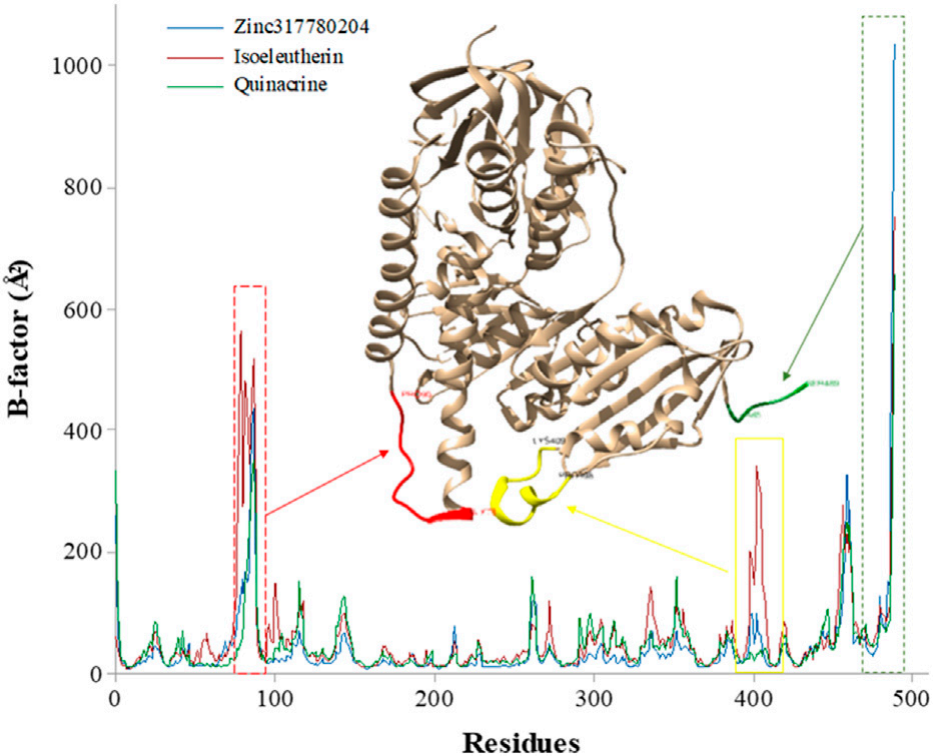
Graph of B-factor (Å) of the enzyme Trypanothione Reductase with the amino acid residues during the MD simulation time (50 ns): isoeleutherin (red line), Zinc317780204 compound (blue line), and quinacrine (green line).

The flexibility of the protein regions in relation to each ligand was evaluated through the graph of B-factor (Figure 7). The greatest fluctuations occurred in regions corresponding to the bands of amino acid residues Asn91-Gly80 (highlighted in red), Glu410-Thr397 (highlighted in yellow) and Ser489 fragment region, highlighted in green, considered the most flexible regions of the enzyme. These residues are found close to the Cys52 and Cys57 active sites and play an important functional role in enzyme inhibition (Verma et al., 2012; Pandey et al., 2016).

Comparing the binding free energy values of the two methods, described in Table 4, both are favorable and stable for complex formation. The calculation of binding free energy allowed for quantifying the affinity between the enzyme-ligand system. When comparing the free energy values obtained through the MM-GB(PB)SA method, it was observed that quinacrine, used as the reference drug in the present study, displayed the lowest value. The compound Zinc317780204 followed, with values lower than those of isoeleutherin, indicating that these compounds may be useful in situations where the reference drug has limitations.

**TABLE 4:** Binding free energies with standard deviation calculated by the MM-GB(PB)SA method.

Complex	ΔG_MMGBSA_ (kcal/mol)	ΔG_MMPBSA_ (kcal/mol)
TR-Isoeleutherin	−16.17 ± 4.21	−16.54 ± 4.46
TR-Zinc317780204	−25.34 ± 2.25	−19.67 ± 3.45
TR—Quinacrine	−48.92 ± 3.87	−25.07 ± 4.50

ΔG_MMGBSA_ (free energy values obtained by molecular mechanics method with generalized born surface area); ΔG_MMPBSA_ (free energy values obtained by molecular mechanics with Poisson-Boltzmann surface area).

Energy decomposition by residue was employed to identify the TR enzyme residues involved in interactions with Zinc317780204, isoeleutherin, and quinacrine, based on the energy contribution calculated by the MMGBSA method for the last 10 ns of MD simulations. Free binding energy values below −1 kcal/mol were set as the criteria for selecting amino acid residues that participated in the most favorable interactions, contributing to ligand stabilization within the complex.

**FIGURE 8:**
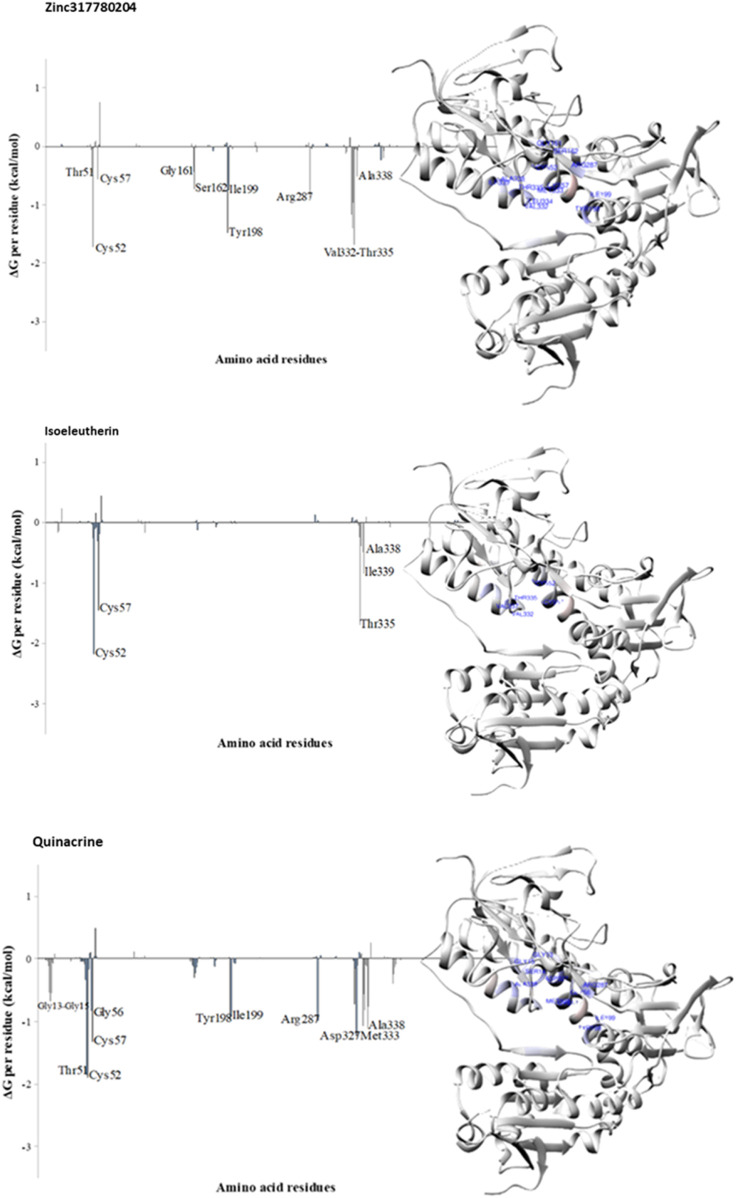
Decomposition of free binding energy per residue (in kcal/mol) obtained with the MMBGSA method for TR-Zinc317780204, TR-Isoeleutherin and TR-Quinacrine. On the right, residues that most contributed to the binding free energy, visualized by CHEWD plugin in the Chimera program, are shown in blue.

The analysis of residue decomposition values reveals those that played a significant role in the total interaction energy and stabilization of the TR-Zinc317780204 (Cys52, Tyr198, Val332, and Thr335), TR-isoeleutherin (Cys52, 57, and Thr335), and TR-Quinacrine (Cys52, Tyr51, Cys57, Arg287, Asp327, Met333, and Ala338) complexes, contributing significantly to the overall free energy of the complex (Figure 8).

## 4 Discussion

The identified chemical constituents of *E. plicata* bulbs, involve several classes of phy-tochemicals, condensed tannins, coumarins, steroids, triterpenoids, anthraquinones and naphthoquinones, being isolated, eleutherol, eleutherin and their respective isomers, isoeleutherol and isoeleutherin, indicative of being the major constituents and markers of the species (Malheiros et al., 2015; Vale et al., 2020).

One study evaluated the EEEp, PDEp and isoeleutherin that were active in another parasite, *Plasmodium falciparum* sensitive to chloroquine (Vale et al., 2020), reinforcing the premise that the antiparasitic activity of *E. plicata* is related to naphthoquinone isoeleutherin. However, it is important to investigate the possible mechanisms involved in these antiparasitic activities.

Regarding the physicochemical properties, all compounds met the criteria of Lipinski’s rule of five, The octanol-water partition coefficient allows to determine the degree of hydrophobicity of the molecules and directly influences the absorption and bioavailability of drugs, substances that present (miLog *p* ≤ 5), are more soluble in organic medium, in this study, all molecules present miLogP within the established parameter, demonstrating that they have a more polar character, capable of dissolving in aqueous and crossing cellular barriers (Lipinski et al., 1997). The compounds had a molecular weight >500 Da, and this may be directly related to the permeability of the substances, since the higher the molecular weight, the more difficult it will be for the compound to permeate biological membranes (Lipinski et al., 1997). The topological polar surface area (TPSA) of each molecule was less than 140 Å, which is justified by the lower bond between hydrogen acceptors and donors, respectively ≤10 and ≤5. Thus, isoeleuterin would be the most promising compound, due to its lower molecular weight and better physicochemical profile for permeability between cell membranes.

In the pharmacokinetic prediction, due to the physicochemical characteristics of the polar compounds, the permeability in MDCK cells (Madin-Darby canine kidney) was low to moderate, when evaluating the permeability in MDCK cells, the active permeability rate of each molecule was analyzed. All molecules have moderate permeability to Caco-2 cells (human colon carcinoma epithelial cells), suggesting that the rate of intestinal absorption is moderate through the passive diffusion mechanism (Chen et al., 2018; Panse and Gerk, 2022). However, isoeleutherin and its studied analogues have been shown to have high absorption in the small intestine through human intestinal absorption analysis. Regarding the permeability of the blood-brain barrier, only isoeleutherin and compound 18 freely cross the blood-brain barrier, probably due to their lower molecular weight and better plasma distribution, due to their lower binding to plasma proteins compared to the other compounds that showed high binding to plasma proteins, so isoeleutherin has a better pharmacokinetic profile of absorption and distribution.

In the present study, the involvement of RT in the leishmanicidal activity of isoeleutherin and analogues was evaluated. TR is an NADPH-dependent flavoprotein disulfide reductase, found in several parasites, including *Leishmania*, which participates in its redox system, being a substitute for the glutathione/glutathione reductase and thioredoxin/thioredoxin reductase systems (Baiocco et al., 2009; Fairlamb and Cerami, 1992]. Naphthoquinones, in the presence of oxygen, are reduced and reoxidized, generating reactive oxygen species that affect the capacity of TR, which is why this target was selected (Ferreira, 2008).

In *Leishmania spp*. and trypanosomatids, the redox balance is carried out by the enzyme TR, which functions as a FAD-dependent disulfide oxidoreductase that catalyzes the reduction of trypanothione [N1, N8-bis-glutathionylspermidine or T (SH)2] as a function of NADPH (Leroux and Krauth-Siegel, 2016). Trypanothione is a dithiol formed by two glutathione molecules linked by a spermidine bridge, it acts as an electron donor in biological reactions, including the elimination of hydroperoxides, being the main thiol in trypanosomatids, assuming the functions of glutathione in other organisms. The return of trypanothione to its reduced active form is carried out by TR, an essential process for the redox balance of the parasite and cell viability, demonstrating the essential character of TR (Tovar et al., 1998). The closest mammalian homologue of TR is the enzyme glutathione reductase (GR), despite their general similarity, TR and GR differ significantly in their thiol binding sites, making it feasible to target TR with chemical entities that do not compromise GR activity (Castro et al., 2018). Over-all, the essential role played by TR in *Leishmania* spp. and its absence in the human host, makes this molecule an attractive target for the development of potential new drugs.

In this work, the leishmanicidal activity was demonstrated, against promastigotes forms of *L*. *amazonensis*, where FDEp showed moderate activity and isoeleutherin was more promise. We also observed the presence of parasites around infected macrophages, requiring further investigation, whether there was interference in the mechanism of phagocytosis or destruction of these cells. Everything indicates that derivatives of *E. plicata*, especially isoeleutherin, have activity on *L*. *amazonensis.*

The synthetic naphthoquinone, LQB-118 caused a concentration-dependent reduction in the number of amastigotes of *L. amazonensis* (Cunha-Junior et al., 2011). The hydroxynaphthoquinone Buparvaquone showed excellent *in vitro* activity against amastigotes of *L. donovani* (Effective dose 50%—ED50 between 0.12 and 0.005 µM) (Croft et al., 1992). The dimeric naphthoquinones 3,3-Bijuglone and 6,6-Dibenzyloxy-3,3-bi-plumbagin, exhibited high toxicity for amastigotes of *L. donovani* (IC_50_ of 15 and 14.2 μg/mL, respectively) (Kaiser et al., 2000).

It is known that structural alterations can interfere with the biological activities of quinones, and eleutherin, isoeleutherin and eleutherol presented different immune responses mediated by helper T-cells (Castro et al., 2021b). Isoeleuterin has a 1,4-naphthoquinone ring with an α-methyl group, selectively stimulating IFNc production by activating transcription of the T-bet gene, thus enhancing Th1-mediated immune responses. While, naphthopy-ran-4-one, eleutherinol, inhibited the production of IFNc and IL-2 during the activation of Th cells, suppressing the transcripts of the cytokine gene. Therefore, chemical modification and chirality of the naphthopyran moiety in isoeleutherin and eleutherinol may be critical for the selective modulation of immune responses mediated by helper T-cells (Hong et al., 2008).

In this study, using peritoneal macrophages from BALB/c mice, the cytotoxic activity of EEEp, its fractions (FDEp, FAEp and FMEp) and isoeleutherin were evaluated, with no cytotoxicity being observed. Gomes et al. (2021), evaluated the cytotoxicity of EEEp, FDMEp and isoeleutherin from *E*. *plicata* in human hepatoma cells (HepG2), after exposure for 24 h, and observed that fractionation reduced cytotoxicity, with isoeleutherin being the least toxic sample. These results suggest the cytotoxicity of EEEp and FDMEp are related to the synergism between eleutherin and isoeleutherin compounds. When administered together they are more active, which may increase toxicity (Vale et al., 2020; Gomes et al., 2021).

Another study using an integrative approach of *in vitro* and *in silico* methodologies, found isoeleutherin was the compound with lowest cytotoxicity in the micronucleus assay and structural changes. Also, there was an increase in the number of important interactions with amino acid residues of the active site, suggesting increased affinity with the Topoisomerase II enzyme, representing a good starting point in the search for new drugs for anticancer therapy (Albuquerque et al., 2023).

## 5 Conclusion

In summary, structural alterations made to isoeleutherin resulted in obtaining a less toxic analogue compound (CP13), as well as obtaining a more promising molecule as a leishmanicidal agent (CP17). Thus, naphthoquinones may represent an important class of molecules for discovering new leishmanicidal therapies.

## Data Availability

The original contributions presented in the study are included in the article/Supplementary Material, further inquiries can be directed to the corresponding author.
